# Silicon nanowires prepared by electron beam evaporation in ultrahigh vacuum

**DOI:** 10.1186/1556-276X-7-243

**Published:** 2012-05-06

**Authors:** Xiangdong Xu, Shibin Li, Yinchuan Wang, Taijun Fan, Yadong Jiang, Long Huang, Qiong He, Tianhong Ao

**Affiliations:** 1 State Key Laboratory of Electronic Thin Films and Integrated Devices, School of Optoelectronic Information, University of Electronic Science and Technology of China (UESTC), Chengdu, 610054, People's Republic of China; 2 College of Chemistry and Molecular Engineering, Peking University, Beijing, 100871, People's Republic of China

**Keywords:** Silicon nanowires, Preparation, Vapor–liquid-solid, Oxide-assisted growth, Ultrahigh vacuum

## Abstract

One-dimensional silicon nanowires (SiNWs) were prepared by electron beam evaporation in ultrahigh vacuum (UHV). The SiNWs can be grown through either vapor–liquid-solid (VLS) or oxide-assisted growth (OAG) mechanism. In VLS growth, SiNWs can be formed on Si surface, not on SiO_2_ surfaces. Moreover, low deposition rate is helpful for producing lateral SiNWs by VLS. But in OAG process, SiNWs can be grown on SiO_2_ surfaces, not on Si surfaces. This work reveals the methods of producing large-scale SiNWs in UHV.

## **Background**

One-dimensional (1D) nanomaterials have stimulated great interest due to their importance in basic academic research and potential technology applications [1,2]. It is widely accepted that 1D nanomaterials not only play vital roles as interconnects and functional units for nanodevices, but also provide opportunities to investigate the dependence of electrical, thermal, and mechanical properties on the dimensionality and size reduction [2-4]. Among all 1D nanomaterials, silicon nanowires (SiNWs) are particularly attractive [5-9] because of the center role of silicon (Si) in semiconductor industry. Moreover, a wealth of traditional knowledge about Si material is helpful for understanding the relationships between its properties and nanostructures. In addition, the mature Si-based technology can be exploited to fabricate future nanodevices.

Many methods have been developed to prepare 1D SiNWs [5-16]. However, most SiNWs were produced in air atmosphere or low vacuum conditions [5-12]. Synthesis of SiNWs in ultrahigh vacuum (UHV) is helpful for obtaining highly pure products, as well as better evaluating their properties and understanding the related mechanisms. Therefore, the growth of SiNWs in UHV has attracted considerable attention recently [13-16]. Schubert et al. reported the preparation of vertical Si nanowhiskers in UHV by molecular beam epitaxy through vapor–liquid-solid (VLS) growth [13]. Similarly, Irrera et al. prepared vertical SiNWs in UHV by electron beam evaporation (EBE) through VLS [14,15]. Differently, Xu et al. introduced a new method to synthesize SiNWs in UHV by EBE through oxide-assisted growth (OAG) mechanism [16].

In this work, we demonstrated the feasibility of producing lateral SiNWs in UHV by EBE through both VLS and OAG mechanisms. The critical factors for 1D nanowire formations were also discussed.

## **Methods**

Si(111) wafers sized 1 × 1 cm^2^ were chosen as the deposition substrates. Before being used, the Si substrates were ultrasonically cleaned in methanol for 15min followed by a dip in a diluted hydrofluoric acid (HF) solution for removing organic contaminations and surface oxides. The SiO_2_/Si(111) substrates were prepared by deposition of SiO_2_ films on Si(111) wafers using a plasma-enhanced chemical vapor deposition system (Orion II, Trion Technology, Clearwater, FL, USA) under the conditions of 13.56MHz, 600W, 0.6Torr, 300°C, and a N_2_O/SiH_4_ flux ratio of 100/150sccm. These SiO_2_/Si(111) substrates were ultrasonically cleaned with successive rinses of acetone and methanol. After having been dried by N_2_ gas, the cleaned substrates were transferred immediately to a UHV EBE system (Balzers ULS 400, Balzers Ltd., Liechtenstein, Switzerland) for depositions at constant rate of 0.02nm/s. The base pressure was 2 × 10^−10^mbar, and the process pressure was maintained at 1 × 10^−7^mbar or below during the depositions.

The as-deposited materials were characterized *exsitu* by tapping mode atomic force microscopy (AFM, Nanoscope III, Veeco Instruments, Inc., Plainview, NY, USA) and scanning electron microscopy (SEM, FEI XL30S-FEG, FEI Company, NE Dawson Creek Drive, Hillsboro, OR, USA), respectively.

## **Results and discussion**

**Figure 1:**
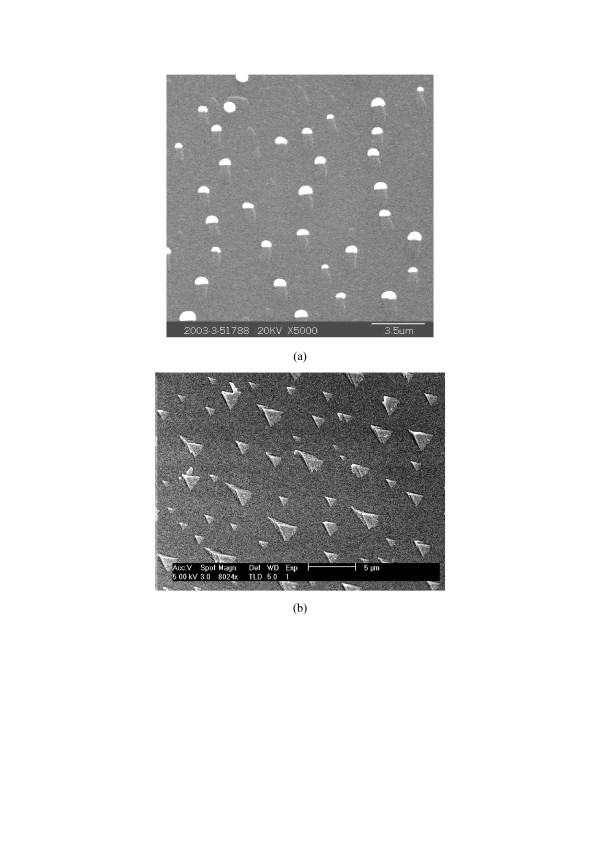
**SEM images of 1D Si nanowires, 2D Si growth and AFM images of AuNPs.** (**a**) SEM image of 1D Si nanowires grown through VLS on Si substrate, (**b**) SEM image of 2D Si growth without Au nano-catalysts on Si substrate, and (**c**) AFM images of AuNPs prepared by similar depositions on SiO_2_ substrate.

Figure 1a shows the morphologies of the materials prepared by successive depositions of 1nm Au at 200°C and 2nm Si at 700°C by EBE onto an HF-treated Si substrate. One can see that numerous Si wires with lengths of 700 to 1,200nm were induced by Au semispherical droplets to grow on the whole substrate surface (Figure 1a). These wires have a low aspect ratio of 2 to 4, and they lie laterally on the Si surface. Locations of Au droplets on the ends of nanowires (Figure 1a) clearly indicate that these SiNWs were grown through VLS, similar to the mechanism in Schubert [13] and Irrera [14,15] processes. But differently, our SiNWs are lateral (Figure 1a), while the others [13-15] are vertical. We believed that the formation of lateral SiNWs results from the competition of two growths. One is the formation of wire structures (1D growth) that are induced by the Au nano-catalysts and tend to grow outwards (vertically) from the substrate surface because of continuous adsorption of Si atoms from the evaporated vapor. Another is the epitaxial (2D) growth of Si that results in the lateral growth of Si on the surface. The epitaxial growth of Si has been proved by our experiment (Figure 1b) that was conducted by Si deposition without Au nano-catalysts. The competition of 1D and 2D growths causes subsequent formation of the structures, as shown in Figure 1a. The failure of producing lateral nanowires by Schubert [13] and Irrera [14,15] is due to high deposition rates (0.05nm/s) in their processes [13-15]. At high deposition rate (like 0.05nm/s in previous others [13-15]), 1D growth is dominant, and thus the main products will be vertical SiNWs. In contrast, both 1D and 2D growths exist at a low deposition rate (like 0.02nm/s in our work), and thus lateral SiNWs will be yielded (Figure 1a). We note that the lateral SiNWs growth was also previously observed under an in-plane solid–liquid-solid process induced by indium catalyst [17].

If a 100-nm-thick SiO_2_/Si(111) was used as the substrate, the case is different after the same Au and Si depositions. Figure 1c reveals that only 0D nanoparticles, but no any 1D nanowires, were formed on the SiO_2_ surface. The large particles in Figure 1c are Au, while the small ones are Si. Figure 1a and c suggest that molten Au-Si alloys are formed on the Si surface, and thus 1D SiNWs growth by VLS is induced (Figure 1a) [5–7,10-13]. But on the SiO_2_ surface, the interactions of Au-O and Si-O between the substrate and arriving species prevent the coalescence of Au and Si. Therefore, no Au-Si alloys can be created on the SiO_2_ surface, and thus no wires are yielded through VLS (Figure 1c). Although Pecora et al. similarly deduced the negative effects of SiO_2_ layer on the growth of SiNWs [18], they ignored the interactions between the substrate and arriving species. We believe that such interactions are critical in physical vapor deposition processes.

**Figure 2:**
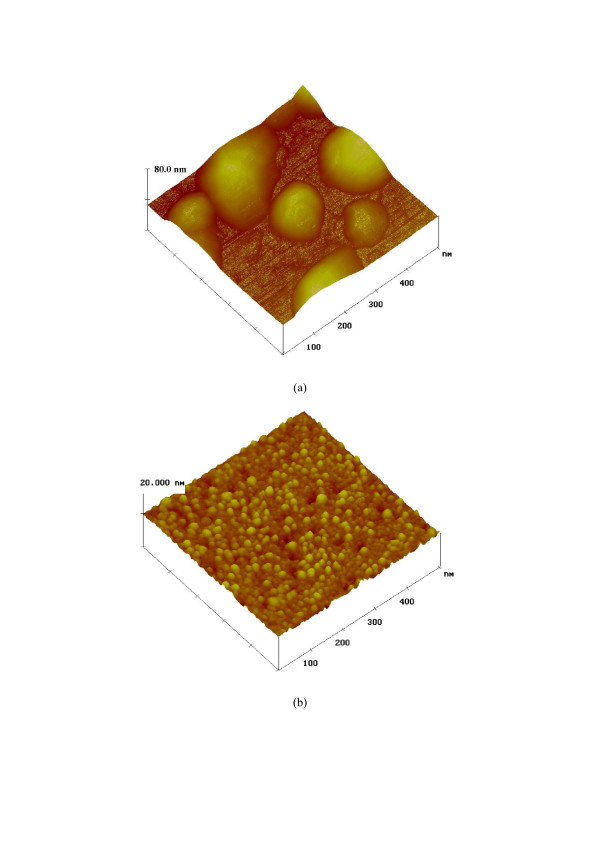
**Three-dimensional AFM images and size distributions of Au particles.** Three-dimensional AFM images of depositions of Au on (**a**) Si and (**b**) SiO_2_ substrates, respectively, and the size distributions of Au particles grown on (**c**) Si and (**d**) SiO_2_ substrates, respectively. Curved lines are the fitting results.

In order to verify the above prediction, we further investigated the growths of Au on Si and SiO_2_ surfaces. The typical AFM results are displayed in Figure 2. Figure 2 indicates that Au nanoparticles (AuNPs) with large diameters of 80 to 180 nm and low density of 5 × 10^8^cm^−2^ were formed when 1nm Au was deposited onto a Si surface (Figure 2a,c). In contrast, AuNPs with small diameters of 8 to 24nm and high particle density of 1.64 × 10^11^cm^−2^ were formed on a SiO_2_ surface under the same conditions (Figure 2b,d). Large and various AuNPs created on Si surface (Figure 2a) suggest weak interactions between the deposited Au species and substrate, while small and uniform AuNPs on SiO_2_ surface (Figure 2b) imply stronger interactions. Figure 2b provides support for the surface O atoms serving as binding sites for controlling the Au growth, as observed by Wahlström et al. [19] and Parker and Campbell [20] on TiO_2_ substrates. We note that this deduction agrees with some phenomena previously reported [7,10]. For example, Cui et al. claimed that AuNPs with small sizes of 5–30 nm pre-prepared on SiO_2_ surface can catalytically induce the growth of 1D SiNWs by chemical vapor deposition [7]. Although the growth was carried out at 440°C, no coalescence of AuNPs occurred, and thus diameter-controlled growth of SiNWs could be achieved [7]. This implies that the agglomeration of Au is prevented by the interaction between Au and SiO_2_. In contrast, Ozaki et al. observed Au agglomeration on SiO_2_ surface after annealing at 500°C [10], suggesting that the interaction between Au and SiO_2_ is weak (6 kJ/mol) [21] and easily damaged. Taking these and our results together, we believe that the strength order of the interaction between Au and substrate is: SiO_2_ > Si. This prediction is further confirmed by the interface energy results: $\gamma_{\text{Au-Si}}{}_{{\text{O}}_{2}}$(1,484mJ/m^2^)>$\gamma_{\text{Au-Si}}{}_{}$(672mJ/m^2^) [22]. Accordingly, Au atoms deposited from vapor on Si surface can easily overcome their weak interactions with the Si substrate, and thus they can migrate and coalesce to form large islands (Figure 2a,c) through diffusion. In contrast, the interaction between Au and SiO_2_ substrates will cause immediate nucleation and growth of Au on where they impinge, thus leading to the formation of AuNPs with smaller sizes and higher density (Figure 2b,d). This is verified by another fact that the Au diffusion coefficient on Si surface ($D_{\text{Au/Si}}{}_{}$=1.9×10^−16^m^2^/s) [23] is significantly larger than that on SiO_2_ surface (($D_{\text{Au/Si}}{}_{{\text{O}}_{2}}$=7×10^−30^m^2^/s)) [24].

**Figure 3:**
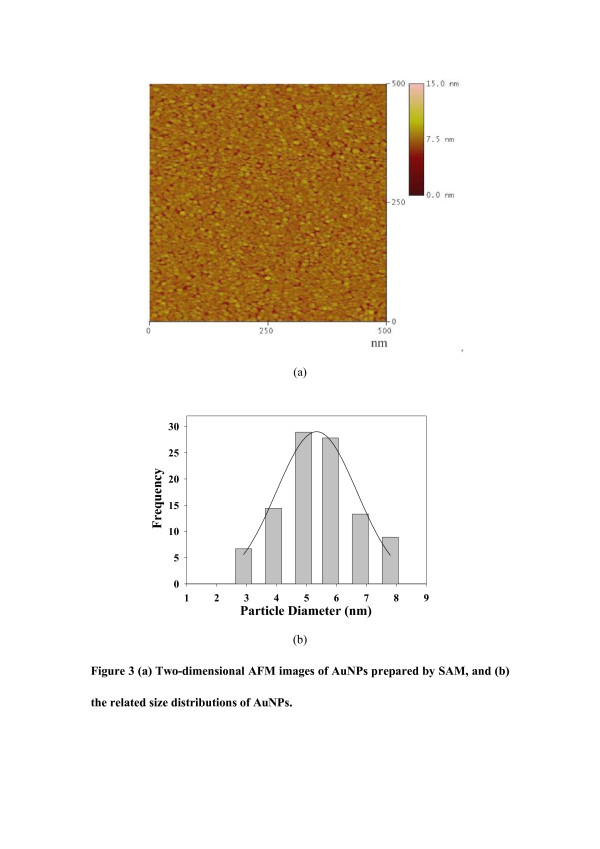
**Two-dimensional AFM images and related size distributions of AuNPs.** (**a**) Two-dimensional AFM images of AuNPs prepared by SAM, and (**b**) the related size distributions of AuNPs.

We further investigated the preparation of AuNPs by self assembly in UHV. In this experiment, we first prepared a 3-mercaptopropyltrimethoxysilane (MPTMS) self-assembled monolayer (SAM) on a Si(111) substrate, and then vaporized Au by EBE in UHV onto the MPTMS surface [25]. Figure 3 reveals that AuNPs with diameters of 3 to 8nm (Figure 3b) and particle density of 1.37 × 10^12^cm^−2^ were formed and distributed uniformly and densely. Smaller and more uniform AuNPs, other than those in Figure 2a and b, were formed on the MPTMS surface (Figure 3a). This is attributed to stronger Au-SR bonds (165kJ/mol) between Au and MPTMS [25], further confirming our prediction about the effects of interaction on the growth.

**Figure 4:**
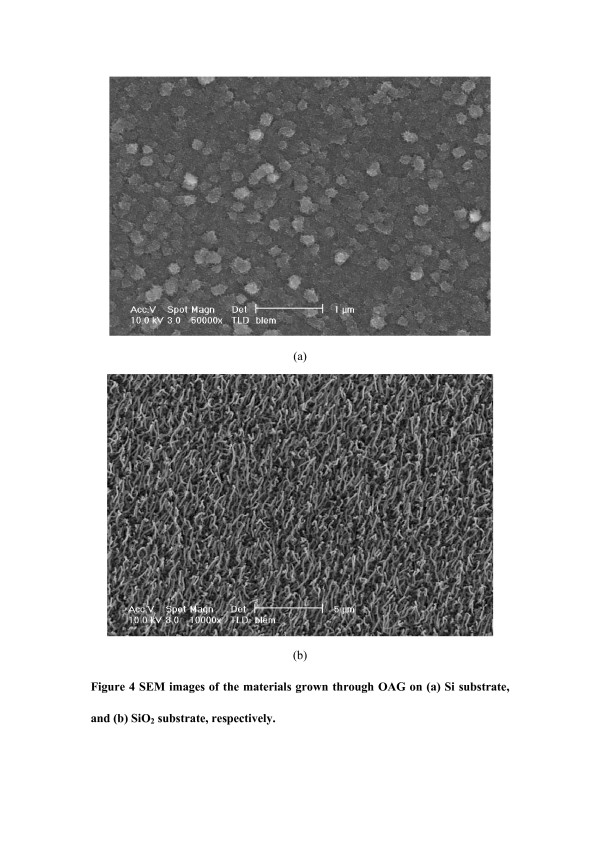
**SEM images of the materials grown through OAG.** On (**a**) Si substrate, and (**b**) SiO_2_ substrate, respectively.

Finally, we investigated the growth of SiNWs through OAG in UHV. In this case, a mixture of Si and SiO_2_ powders with a molar ratio of 2:1 was used as the source that was evaporated by 7keV electron beam. Very interestingly, no any wire structures were formed at 700°C on the Si surface (Figure 4a), and reversely, large-scale 1D SiNWs with a high aspect ratio of 10 to 50 and lengths ranging from 1 to 4μm were produced through OAG on the SiO_2_ surface under the same conditions (Figure 4b). This phenomenon (Figure 4) is completely different from that in VLS process (Figure 1). According to our previous study [16], SiNWs (Figure 4b) are grown by OAG via a disproportionation reaction of 2SiO → Si + SiO_2_. The SiO intermediates are created by electron beam bombardment on the mixed Si and SiO_2_ and play an important role in the SiNWs formation [16]. Firstly, SiO can be bonded to the SiO_2_ surface through the overlap between the empty Si π_2p_* orbits and the lone pair filled O 2p orbits (formation of an intermolecular Si ← O donor-acceptor bond) [26], which contributes the generation of stable nuclei for the growth of SiNWs. Secondly, disproportionation of SiO creates “inert” SiO_2_ sheath, which saturates the surface dangling bonds of the Si core in the lateral direction and only allows 1D growth (Figure 4b). However, on the Si surface, the interaction of Si-Si (310kJ/mol) between the Si substrate and deposited Si species is so strong that the arriving species will be pushed to immediately anchor and freeze on where they are deposited. This negatively disturbs the disproportionation reaction of SiO, and the nanowire formation will be thus inhibited. Therefore, the growth processes for VLS and OAG are completely different, so that suitable substrates for 1D SiNWs growth by these two mechanisms are distinct. Our results (Figures 1a and 4b) clearly demonstrate that large-scale 1D SiNWs can be produced by EBE in UHV.

## **Conclusions**

Large-scale SiNWs were successfully prepared by EBE in UHV through both VLS and OAG mechanisms. In VLS growth, Au-Si alloys created on the HF-treated Si substrates catalytically induce the growth of 1D SiNWs at 700°C. However, the Au-O and Si-O interactions inhibit the formation of Au-Si alloys, and thus no SiNWs can be grown through VLS on the SiO_2_ surfaces. In OAG process, the strong interactions of Si-Si between the Si substrates and deposited Si species prevent the formation of 1D SiNWs. In contrast, SiO_2_ surfaces provide suitable interactions so that SiNWs can be grown at 700°C through OAG on such substrates. This work reveals the methods to produce 1D SiNWs by EBE in UHV.

## Abbreviations

1D = One-dimensional; 2D = Two-dimensional; AFM = Atomic force microscopy; AuNPs = Au nanoparticles; EBE = Electron beam evaporation; OAG = Oxide-assisted growth; SEM = Scanning electron microscopy; Si = Silicon; SiNWs = Silicon nanowires; UHV = Ultrahigh vacuum; VLS = Vapor–liquid-solid.

## **Competing interests**

The authors declare that they have no competing interests.

## **Authors' contributions**

XDX carried out the experiments and wrote the manuscript. SBL participated the writing. YCW and TJF participated part of the experiments. YDJ participated the writing. LH, QH and THA summarized the experimental data. All authors read and approved the final manuscript.
